# Dopamine-β-Hydroxylase Activity and Levels of Its Cofactors and Other Biochemical Parameters in the Serum of Arsenicosis Patients of Bangladesh

**Published:** 2014-03

**Authors:** M. Khalilur Rahman, M. Iqbal Choudhary, M. Arif, M. Monzur Morshed

**Affiliations:** 1 Department of Biochemistry and Molecular Biology, University of Dhaka, Dhaka-1000, Bangladesh;; 2 Dr. Panjwani Center for Molecular Medicine and Drug Research, International Center for Chemical and Biological Sciences, University of Karachi, Karachi 75270, Pakistan

**Keywords:** Arsenicosis, Drinking-water, Dopamine-β-Hydroxylase, Norepinephrine, Serum

## Abstract

Dopamine-β-hydroxylase (DBH) is a neurotransmitter (catecholamine)-mediating enzyme, which catalyzes the formation of norepinephrine from dopamine. The levels of DBH activity, its coenzyme (ascorbic acid) and cofactor (Cu^++^) and other biochemical parameters were measured in the serum of 32 arsenicosis patients of Bangladesh at three different age groups, namely, group 1 (10–18 years, 9 patients), group 2 (19–40 years, 14 patients) and group 3 (41–70 years, 9 patients) of the locality of Stadium Para of Meherpur district of Bangladesh. The values were compared with the same number of age-matched normal healthy individuals of the respective group. DBH activity was markedly decreased in the patients of group 1 as compared to that of the normal healthy people. The activities of DBH were decreased to lesser extents for the other two age groups. The total protein contents in the serum of arsenicosis patients were also significantly low as compared to that in the age-matched control groups. The levels of ascorbic acid and copper were found to be decreased in the serum of arsenicosis patients. The serum glucose levels were elevated in arsenicosis patients, as compared to that of the respective healthy controls. Other parameters, such as zinc and vitamin A levels were also decreased in the serum of arsenicosis patients. It was evident from the results of drinking of the arsenic contaminated water of shallow tube wells that the levels of DBH activity decreased significantly as compared to the control healthy persons. The levels of proteins, ascorbic acid, copper, zinc and vitamin A were decreased in the serum of people drinking the arsenic contaminated tube wells water as compared to that in the control healthy people with the exception that the levels of glucose were elevated in the serum of these patients. The pathophysiological significance of the results could be correlated with the decreased in proteins and that in DBH activities as DBH deficiency is characterized by lack of sympathetic noradrenergic function.The general physiologic findings of autonomic function indicate that complete DBH deficiency include minimal or absent plasma norepinephrine and epinephrine.

## INTRODUCTION

Arsenicosis has emerged as a cynosure and devastating public health problem in many countries of the World, including Bangladesh. It posed a considerable threat to Bangladesh as more than 65 million people from 49 districts of Bangladesh are at a risk of arsenic poisoning and nearly 220,000 people have already displaying symptoms of arsenicosis. The world Bank estimated about 18 million people of Bangladesh have already been exposed to drinking arsenic contaminated ground water (1). Every year the affected areas and hence the number of affected people are being increased by nearly 20% in Bangladesh (2). Bangladesh is geographically adjacent to West Bengal of India having similar aquifers and socioeconomic background. The alluvial formation of Bangladesh has close geographical similarity to that encountered in West Bengal of India. In Bangladesh the ground water has been withdrawn vigorously through deep tube-wells for irrigation purposes particularly during drought and winter-season (3). According to WHO, in Bangladesh and India, the permissible limit of arsenic in drinking water is 50 µg/L (4). Under this guideline, the concentration of 10 µg/L arsenic in water is safe and tolerable.

But in most of the arsenic contaminated water of shallow tube-wells contain the much higher amounts of arsenic which were taken by the human beings.

The clinical manifestations of arsenic toxicity were categorized as (5, 6): Initial stage which includes melanosis, keratosis, conjunctivitis, bronchitis, gastroenteritis; the symptoms and effects of secondary stage are depigmentation (leukomelanosis), hyperkeratosis, non-pitting edema, peripheral neuropathy, neuropathy (early stage), hepatomegaly (early stage) and the tertiary stage includes neuropathy (last stage), hepatomegaly (last stage), gangrene, cancer and kidney failure. The chronic toxicity of arsenic is best discussed in terms of the organ system affected, including the sympathetic and parasympathetic nervous systems, the skin, liver , cardiovascular system and respiratory tract.

The usual symptoms of arsenicosis (7): The symptoms of arsenic toxicity may take 8–14 years to be manifested in a person’s body after the patient starts drinking arsenic contaminated water. This period differs from patient to patient depending on the quantity/volume of arsenic ingested, nutritional status of the person, immunity level of the individual and the total time-period of arsenic ingested. The apparent symptoms of arsenicosis may be said to have manifested themselves as melanosis and keratosis mainly.

Dopamine-β-hydroxylase [3, 4-dihydoxyphenylethylamine, ascorbate:oxygen oxidoreductase (3-hydroxylating), EC 1.14.17.1; abbreviated DBH] is the enzyme responsible for the biosynthesis of catecholamine neurotransmitter, norepinephrine from dopamine (8, 9), ultimately leading to the formation of epinephrine by the enzyme - Phenylethanolamine-N-Methyl Transferase (PNMT) in the mammalian tissues and serum. The product, norepinephrine, is important biochemically and pharmacologically, because the monoamines are important intracellular messengers, such as neurotransmitters and hormones and involve in the regulation of neuronal functions, behavior and emotion of higher animals. The DBH is a copper (Cu^++^) and ascorbic acid (Vitamin C) dependent enzyme and thus DBH is a mixed function copper containing oxygenase (10). The enzyme activity is stimulated by the addition of dicarboxylic acids such as fumaric acid.

DBH is an intraneuronal enzyme in the sympathetic nervous system and enzyme activity was found in the adrenal medulla (11), in the brain (12) and in various sympathetically innervated organs, such as heart (13, 14). In humans and laboratory animals, DBH has been measured in plasma and serum (15, 16), in cerebrospinal fluid (CSF) (17). DBH being one of the key neurotransmitter mediating enzymes has drawn much attention from clinical and pharmacological investigator as a possible index of the sympathetic nervous functions. Deficiency of dopamine results in various neurological disorders, mainly Parkinson’s disease (18, 19). A significant increase in DBH activity occurs in pheochromocytoma (20). It has been also reported that DBH deficiency resulted in poor cardiovascular regulations (21) and genetic disorder was also associated with its deficiency (22). The first symptoms of DBH deficiency had been often startedduring a complicated perinatal period with hypotension, muscle hypotonia, hypothermia and hypoglycemia (23).

The tertiary stage of arsenic toxicity in human causes the neuropathy (5, 6) which may alter the activities or actions of central and peripheral nervous systems due to the changes in the activities of all or some of the neurotransmitters-mediating enzymes, like, Aromatic L-Amino Acid decarboxylase (AADC), DBH and PNMT, and their neurotransmitter-products (dopamine, serotonin, norepinephrine and epinephrine). Long term exposure to arsenic is related to vitamin A deficiency which is related to heart disease and night blindness (24). In this study, we measure the DBH activities along with its cofactors and other biochemical parameters in the serum of arsenicosis patients of Bangladesh at different physiological stages.

## MATERIALS AND METHODS

### Arsenic Contaminated Water, Arsenicosis Patients and Blood Collection

The water of many shallow tubewells of the locality of Stadium Para of Meherpur District of Bangladesh was contaminated with arsenic in the ranges greater than the admissible amounts as recommended by the WHO (4). Fifty two water samples (each sample contained 10.0 ml of water) were collected from the study venue and they were analyzed by Flame Atomic Absorption Spectrophotometer (AAS, Pye-Unicam SP9).

It was very difficult to get and to select enough arsenicosis samples. A total 32 arsenicosis patients from the study area could be selected; these patients had the following clinical manifestations (number of the cases and % of total cases which also included the overlapping clinical manifestations are mentioned in the parentheses): Melanosis [27; 84.3], keratosis [23; 71.8], hyperkeratosis [08; 25.0], abdominal pain [04; 12.5], diarrhea [07; 21.8], nausea [03; 9.3], edema [02; 6.3], anorexia [04; 12.5] and conjunctivitis [01; 3.1]. The blood samples were collected (5–10 ml from each patients) from the arsenicosis patients by venipuncture; then the serum was collected from the each sample of the clotted blood. The total samples were placed into three groups on the basis of the age of the patients. There were 9, 14 and 9 samples of the age groups of 10–18 years, 20–40 years and 45–70 years, respectively. The control blood samples were collected from the healthy individuals of same number of samples as mention in each age group, respectively. They had no history of drinking the arsenic contaminated water, nor they had any clinical manifestations of arsenicosis. All the serum samples were stored in the deep freeze (-20°C) until use.

### The Assay of DBH Activity and Other Biochemical Parameters

The DBH activity was assayed by using the simple and rapid method of Kato *et al*. (19). For this assay Catalase, N-Ethylmaleimide, Octopamine, Ascorbic Acid, Sodium Metaperiodate and Sodium Thiosulfate, DOWEX-50W-X4(H^+^, 200 – 400 mesh) were obtained from Sigma Chem. Co. (St. Louis, Mo. 6317, U.S.A.); Tyramine.HCl and fumaric acid were from Calbiochem., San Diego, Calif. 92112, U.S.A.; Pargyline. HCl was from Abbot Lab., North Chicago,III, 600064, U.S.A. The DOWEX-50W-X4(H^+^, 200 – 400 mesh) was activated by cyclic washing with 1 N HCl and 1 M NaOH and finally it was equilibrated with 1 M Na-acetate buffer, pH 5.0 and stored in the same buffer.

The assay method of DBH was simple, rapid and specific for measurement of nmol levels of the enzyme. DBH can also hydroxylate tyramine to octopamine , so tyramine is usually used as substrate for DBH instead of dopamine. The principle of DBH assay is based on the enzymatic conversion of tyramine to octopamine. The same volume of boiled enzyme was used as blank and 4.0 nmoles of octopamine was used in another blank incubation mixture as internal standard.

The product octopamine was isolated by using small column containing 0.2 ml of activated DOWEX-50W- X4(H^+^, 200 – 400 mesh) resin. Then the adsorbed octopamine was eluted with 1.0 ml of 3 N NH_4_OH. The eluted octopamine was converted into p-hydroxybenzaldehyde adding 10µl of 2% NaIO_4_ solution. The excess NaIO_4_ was reduced by adding 10 µl of 10% Na_2_S_2_O_3_ solution.

The solution was extracted with 5.0 ml ethyl ether, the ether phase was again extracted with one ml of 3 N NH_4_OH for the measurements of the absorbance at 330 nm using the experimental, blank and internal standard samples. The absolute amount of octopamine formed was calculated using the following:

$$\text{Octopamine}\;\text{formed}\;(\text{nmoles})=\frac{\left(\text{Absorbance of experimental})–(\text{Absorbance of blank}\right)}{\left(\text{Absorbance of internal standard})–(\text{Absorbance of bank}\right)}\times \;2.0$$

The endogenous inhibitors that interfere with the assay of DBH in vitro were inactivated by adding excess N-methylmaleimide.

Cu^++^ and Zn^++^ in the serum were determined by the procedure using an Atomic Absorption Spectrophotometer (AAS, Pye-Unicam SP9). The estimation of ascorbic acid in the serum was done by the colorimetric ascorbic acid assay kit (BioVision Inc., CA, USA) immediately after the collection of the samples. The estimation of total protein in the serum was also by determined with the protein assay reagent (Bio-Rad aboratories, CA, USA) using the bovine serum albumin as standard. The serum glucose was measured by glucose oxidase method (RANDOX Laboratories, Antrim, UK). Vitamin A was determined in the serum by the HPLC Method (24).

### Statistical Analysis

All the data were analyzed using the Statistical Package for Social Sciences (SPSS) (version 11.0 for Windows, SPSS Inc., Chicago, U.S.A.). Student t-test (two-tailed) was used to evaluate statistical differences between the two study groups. A P-value of ≥0.05 was the criterion for a statistically significant difference. Microsoft Excel and Graph Pad Prism 4.0 (U.S.A.) were used for statistical analyses and graphics. Data were expressed as mean and standard Deviation (± SD).

## RESULTS

### The Level of Arsenic in the Tubewell Water of Arsenic Contaminated Area of Stadium Para of Meherpur District of Bangladesh

**Figure 1:**
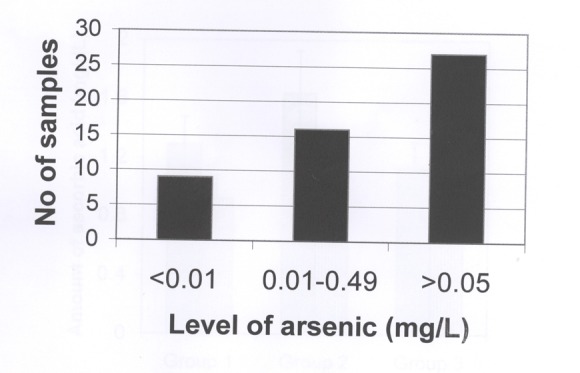
Levels of arsenic in the water of 52 tubewells which the arsenicosis patients had taken and used for more than 10 years of their lives. The location of the tubewells were at Stadium Para of Meherpur district of Bangladesh. Fifty two arsenic contaminated water samples were tested and noted their arsenic concentrations were noted as <0.01 mg/liter, in the ranges of 0.01–0.49 mg/liter and >0.05 mg/liter are shown in Figure 1. Nine samples had the concentrations of less than 0.01; 16 samples contained the arsenic in the ranges of 0.01–0.49 and the remaining 27 water samples had more than 0.05 mg of arsenic per liter of arsenic contaminated water.

Arsenic levels in the tubewells water were measured by Flame Atomic Absorption Spectrophotometer (AAS) in the Soil Resources Development Institute, Farmgate, Dhaka. Figure 1 shows the distributions ranges of arsenic concentrations (mg/L) in the total of 52 arsenic contaminated water- samples from different tubewells; out of these samples, 9 samples (17.3%) contained <0.01 mg/L of arsenic, whereas the concentrations of arsenic in 16 samples (30.75%) were in the ranges of 0.01–0.049mg/L, the rest of the samples, i.e., 27 samples (52%) had shown the arsenic concentrations of more than 0.05 mg/L. The arsenicosis patients of the present study had drunk and used this water for the last 5–10 years of their lives. It might be mentioned here that the manifestations of arsenicosis need at least 5–6 years duration of drinking of the arsenic contaminated water.

### The Dopamine-β-Hydroxylase (DBH) Activity in the Serum of Normal and Arsenicosis Patients of the Three Different Age Groups

Table 1 shows the DBH activity of human arsenicosis patients of three different age groups, namely, Group 1 (10–18 years, 9 samples), Group 2 (20–40 years, 14 samples) and Group 3 (45–70 years, 9 samples). The DBH activities (nmol/min/ml of serum) were significantly decreased (*p*<0.001, *p*<0.003 and *p*<0.007, respectively) in the serum of arsenicosis patients of all the groups as compared to that in the age-matched control groups (Table 1). The results showed that the DBH activities were largely affected in the young arsenicosis patients than that in the other two Groups.

**Table 1:** The DBH activity in the serum of arsenicosis patients in three different age group

Age Groups (Years)	No. of Samples	DBH activity (nmol/min/ml of serum)^a^	
		Normal	Patient

Group 1 (10-18)	9	24.73 ± 5.20	11.13 ± 3.34^b^
Group 2 (20-40)	14	31.98 ± 4.51	23.16 ± 4.96^c^
Group 3 (45-70)	9	30.69 ± 6.66	25.4 ± 5.2^d^

a MEAN ± SD;

b *p*<0.001;

c *p*<0.003;

d *p*<0.07.

### The Specific Activity of DBH in the Serum of Arsenicosis Patients in Three Different Age Groups

Table 2 shows the specific activities of DBH in the serum of human arsenicosis patients at three different age groups of Group 1, 2 and 3 as mentioned above. The specific activities of DBH (pmol/min/mg of serum protein) were significantly low in the serum of arsenicosis patients of groups 1 (*p*<0.001) and 2 (*p*<0.003) as compared to the age-matched control groups. But the specific activities were not significantly changed in group 3. The results indicated that in the young arsenicosis patients, serum DBH activities were much more affected than the adult stages of life as in Group 3, there were no differences between the specific activities of the arsenicosis patients as compared to the age-matched healthy control. The reason might be the contents of arsenic in the water and the duration of ingestion.

**Table 2:** Specific activity of DBH in the serum of arsenicosis patients in three different age groups

Age Groups (Years)	No. of Samples	Specific activities of DBH (pmol/men/mg protein)^a^	
		Normal	Arsenicosis Patient

Group 1 (10-18)	9	376.4 ± 79.6	190.2 ± 57.0^b^
Group 2 (20-40)	14	427.5 ± 60.2	348.8 ± 74.7^c^
Group 3 (45-70)	9	399.6 ± 86.7	415.7 ± 85.1^d^

a Values are MEAN ± SD;

b *p*<0.001;

c *p*<0.003;

d *p*=not significant.

### The Total Protein Contents in the Serum of Three Different Age Groups of Arsenicosis Patients

Table 3 shows the total protein contents in the serum of three different age groups of Bangladeshi arsenicosis patients. The total protein contents (g/dl of serum) in the serum of arsenicosis patients were also significantly low (*p*<0.05 for Group 1, *p*<0.05 for Group 2 and *p*<0.001 for Group 3) as compared to that in the age-matched control groups as compared to that of control healthy groups. The results indicated that the arsenicosis not only affected the DBH levels in the serum of various age groups of people, it also affected the various proteins of the serum of the patients.

**Table 3:** The total protein content in the serum of three different age groups of arsenicosis patients and normal healthy individual

Age Groups (Years)	No. of Samples	Specific activities of DBH (pmol/min/mg protein)^a^	
		Normal	Arsenicosis Patient

Group 1 (10-18)	9	6.57 ± 0.73	5.85 ± 0.47^b^
Group 2 (20-40)	14	7.48 ± 0.56	6.64 ± 0.63^c^
Group 3 (45-70)	9	7.68 ± 0.87	6.11 ± 0.40^d^

a MEAN±SD;

b *p*<0.02 (Significant, *p*≤0.05);

c *p*<0.05 (Moderately significant, *p*<0.05);

d *p*<0.005 (Highly significant, *p*<0.001).

### Ascorbic Acid, Copper, Zinc, vitamin A, Levels in the Serum of Various Arsenicosis Patients

**Figure 2:**
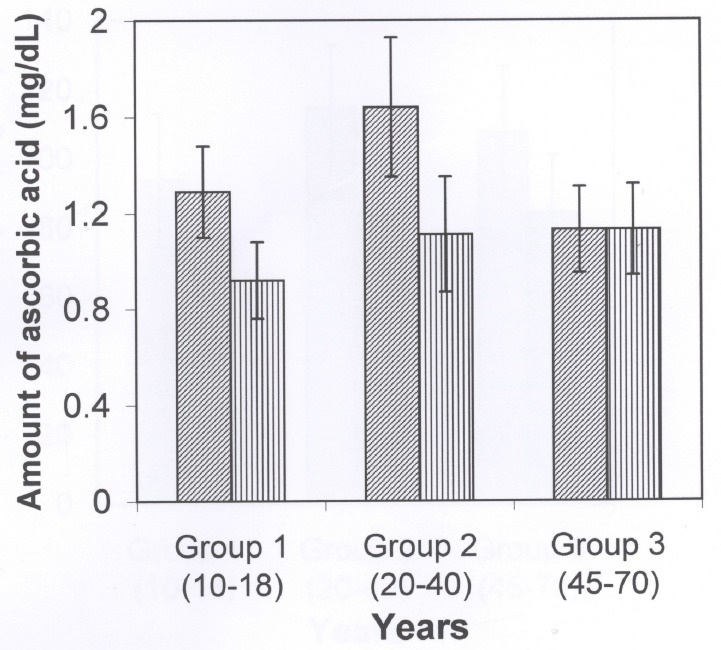
The ascorbic acid contents in the serum of three different age groups(group 1, 10–18 years of age; group 2, 20–40 years of age and group 3, 45–70 years of age) of arsenicosis and for each group age-matched normal healthy controls. The statistical analysis showed that the serum of arsenicosis patients had the low contents of ascorbic acid and the p-values were as follows: *p<0.001 for Group 1; *p<0.002 for group 2 and *p<0.05 for group 3. There were 9 arsenicosis serum samples for each of groups 1 and 2, but there were 14 samples for group 3. The samples sizes were varied because of unavailability of the arsenicosis patients. The ascorbic acid contents were assayed immediately after the samples were collected from the volunteers patients. The normal healthy control individuals had no history of neurological diseases nor did they had taken any arsenic contaminated water in their life time.

**Figure 3:**
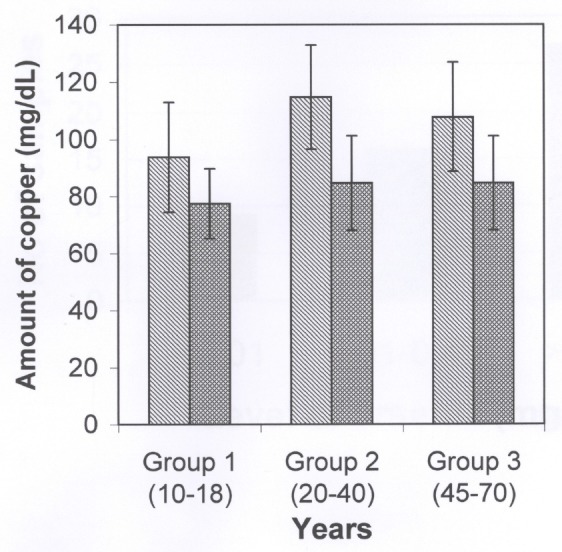
The copper contents in the serum of three different age groups of arsenicosis patients and the normal healthy controls. The statistical analysis showed that there were decreased in the copper contents in all of the three groups as compared to the age-matched normal healthy control groups. The *p*-values were <0.05, <0.001 and <0.001 for group 1, group 2 and group 3, repectively. In this figure also the normal healthy control groups had no history of any neurological diseases nor did they used and took any arsenic contaminated water in their whole lives.

Figure 2 shows the ascorbic acid levels in the serum of three different age groups of arsenicosis patients and the age-matched normal healthy controls. The values were obtained by assaying this coenzyme of DBH which is also an important water soluble vitamin, immediately after the samples were obtained and values were expressed as mg/dl of serum. The results showed that the contents of ascorbic acid were significantly low (*p*<0.001 for group 1 and group 2; *p*<0.05 for group 3) in the all of the arsenicosis samples of serum as compared to that of respective healthy control groups. The other important cofactor of DBH is copper which was also measured in all the serum samples of arsenicosis patients and expressed as mg/dl of serum. As shown in Figure 3, the levels of this cofactor were decreased (*p*<0.05 for group 1; *p*<0.001 for group 2 and *p*<0.05 for group 3) in all the patients of groups 1, 2 and 3 as compared to that in the healthy people of the respective group.

The concentrations (μg/dl of serum) of zinc, important trace element necessary for mammals for the development of congenital organs and which is also an integral parts of many enzymes, were also measured in the serum of all the arsenicosis patients of Groups 1, 2 and 3; the values were found to be 74.1 ± 14.76, 89.6 ± 14.18 and 85.8 ± 11.18, respectively as compared to that in the respective healthy control groups (98.11 ± 20.5, 108.5 ± 13.6 and 94.5 ± 13.87). The results showed that the values were significantly decreased (*p*<0.05 for groups 1 and 2; *p*<0.02 for group 3) in the serum of arsenicosis patients as compared to the age-matched healthy control people.

Table 4 shows the glucose contents in the serum of three different age groups of arsenicosis patients with the age-matched normal healthy controls. The amount of glucose (mg/dl of serum) were significantly high (*p*<0.05 for group 1; *p*<0.04 for group 2 and *p*<0.05 for group 3) in the serum of the arsenicosis patients of all groups, the values were 104.8 ± 15.5 for group 1, as compared to that of the respective age-matched control group.

**Table 4:** The glucose content in the serum of three different age groups of normal individuals and arsenicosis patients

Age Groups (Years)	No. of Samples	Specific activities of DBH (pmol/min/mg protein)^a^	
		Normal	Arsenicosis Patient

Group 1 (10-18)	9	88.3 ± 10.5	104.8 ± 15.5^b^
Group 2 (20-40)	14	109.0 ± 9.85	128.0 ± 12.4^c^
Group 3 (45-70)	9	179.2 ± 39.2	202.0 ± 16.19^d^

a MEAN ± SD;

b *p*<0.0.05 (Significant 0, *p*≤0.05);

c *p*<0.04 (Moderately significant, *p*<0.05);

d *p*<0.051 (Highly significant, *p*<0.001).

In our studies, it was found that vitamin A levels (µg/dl of serum) were also decreased (*p*<0.05 for groups 1 and 2; *p*<0.07 for group 3) in the serum of arsenicosis patients of all groups (35.2 ± 10.16 for group 1, 45.58 ± 9.69 for group 2 and 48.07 ± 10.1 for group 3) as compared to that in the age-matched control groups (48.96 ± 10.0, 61.2 ± 8.62 and 57.02 ± 13.8, respectively).

## DISCUSSION

Arsenicosis has emerged as a serious and most devastating public health problem in Bangladesh.

According to BACS (Bangladesh Center for Advanced Study) more than 65 million people across the country are at risk of arsenic poisoning (1). The arsenic contamination is caused by the drinking and by the use of house hold works water from theshallow tubewells of the localities. This problem is not new in Bangladesh now, it was also found in Taiwan, West Bengal of India, Thailand, U.S.A., Japan and some other countries (2). But the problem is much serious in Bangladesh and it is now considered as national priority problem to mitigate the arsenic toxicity.

DBH being one of the key neurotransmitter mediating enzymes has drawn much attention from clinical and pharmacological investigator as a possible index of the sympathetic nervous functions. We have studied the developmental changes of DBH in various developmental stages using Long-Evans rats of various age groups of 1 week-, 2 weeks-, 5 weeks-, 8 weeks-, 12 weeks-, 15 weeks- and 27 weeks-old. We observed that there were peak activities of DBH in the brain- and peripheral-tissues and serum of rats at 4-5-weeks of age. The peak activities resulted as the rapid nerve maturation takes place at these young stages of life (16). Deficiency of dopamine results in various neurological disorders, mainly Parkinson’s disease (18, 19). A significant increase in DBH activity occurs in pheochromocytoma (20). It has been also reported that DBH deficiency resulted in poor cardiovascular regulations (21) and genetic disorder was also associated with its deficiency (22). The first symptoms of DBH deficiency had been often started during a complicated perinatal period with hypotension, muscle hypotonia, hypothermia and hypoglycemia (23).

The tertiary stage of arsenic toxicity in human causes the neuropathy (5, 6) which may alter the activities or actions of central and peripheral nervous systems due to the changes in the activities of all or some of the neurotransmitters-mediating enzymes, like, Aromatic L-Amino Acid decarboxylase (AADC), DBH and PNMT, and their neurotransmitter-products (dopamine, serotonin, norepinephrine and epinephrine). Long term exposure to arsenic is related to vitamin A deficiency which is related to heart disease and night blindness (24). The present studies were under taken to measure the levels of DBH , its cofactors (vitamin C and copper) and other biochemical parameters (protein, glucose, zinc, vitamin A) at various age groups of arsenicosis patients. These are very important neurochemical and clinical parameters linked to DBH, malnutrition, congenital diseases at various physiological stages of humans including the young, adult and old-age stages. To develop arsenicosis, it takes about 5-6 years of exposure to arsenic contaminated drinking and using water. Therefore, the arsenicosis blood samples were found from the age of 10 years of age. Vitamin C and copper are the cofactors of DBH and neurochemically very important to link to DBH activities; Total proteins, vitamin A, glucose and zinc are very important parameters relating to nervous functions, skin-diseases, diabetes and congenital malfunctions. Therefore, levels of these parameters were measured in the arsenicosis serum.

Fifty two arsenic contaminated water samples of shallow tubewell’s were collected from the Stadium Para village of Meherpur District of Bangladesh. The tubewells were 15–20 years old and the average dept was 65 feet. The people of this locality were drinking and using this water for the last 5–10 years. Out of these 52 samples, 9 samples (17.3%) contained <0.01mg/L of Arsenic, whereas the concentrations of arsenic in 16 samples (30.75%) were in the ranges of 0.01 –0.049mg/L, the rest of the samples, i.e., 27 samples (52%) had shown the arsenic concentrations of more than 0.05 mg/L. After careful considerations and screenings of arsenicosis symptoms (melanosis, keratosis, hyperkeratosis, abdominal pain, diarrhea, nausea, edema and anorexia, gangrene, etc.), serum from 32 patients were used in this study.

DBH is the most important enzyme in the catecholeamine biosynthesis. It is a copper containing glycoprotein consisting of four identical subunits and catalyzes the oxidation of dopamine to peripheral sympathetic nerves and in the adrenal medulla and secreted into blood by a process of exocytosis, resulting a very small amount of DBH is found in the human serum. DBH activity in the serum is considered as a possible index of sympathetic nervous functions. We have shown in our previous studies that the serum DBH activity had altered in various neurological diseases (18). Nagatsu *et al.* had reported (17) a decrease in the DBH level in the cerebrospinal fluid and brain of patients with Parkinson’s disease. In our another study, we have shown that DBH activity was decreased significantly in the Bangladeshi neurological patients of all the age groups (20). In our another studies, we have measured the various catecholamines and indoleamines mediating enzymes in Parkinson’s disease and other extrapyramidal diseases; it was observed that DBH activities were decreased significantly in all these diseases (18). We also produced severe arsenic toxicity in various central and peripheral tissues and serum of mice by feeding very low dosage of sodium arsenite in mice models. The data of these research will be published elsewhere, but we have seen that DBH activities were found to be increased in liver and kidney and slightly increased in heart whereas remained unchanged in spleen. On the other hand, DBH activities were found to be decreased in serum and whole brains of arsenic contaminated mice. Our present data are quite in agreement with our clinical data on rat and human DBH studies.

The product of DBH is not only a neurotransmitter, but also a hormone, which regulate the emotion, behavior,sex, mood and sleep. So, it is very important in biochemical, pharmacological as well as neurochemical point of view. It is the fact that the the measurement procedure of DBH activity is very difficult as it is present in a very small amount. The severity of arsenic poisoning, various biochemical and nutritional changes and the largely affected young age group (10–18 years) tempted us to carry out this study of the neuronal activities of arsenicosis patients.

For this research, a total of 32 serum samples from 32 arsenicosis patients (both male and female) of three different age groups , namely, 9 samples of 10 – 18 years of age (Group 1), 14 samples of 20–40 years of age (Group 2) and 9 samples of 45 – 70 years of age (Group 3) were subjected to the assay of DBH activities with a total of 32 normal healthy controls, who were divided into similar age-matched 3 groups to serve as controls. The coenzyme, cofactors (ascorbic acid and Cu^++^) and several other biochemical parameters were also determined by using the samples of both the arsenicosis patients and age-matched normal healthy control subjects for comparative studies.

The DBH activities from these serum samples were measured by the sensitive method proposed by Kato *et al.* (19). The DBH activity was found to be decreased in the arsenic patients of all age groups as compared to the age-matched control groups. A significant decrease (*p*<0.001) in DBH activity was found at the age group of 10–18 years. The DBH enzyme activity was 11.13 ± 3.34 nmol/min/ml of ml of serum in the arsenicosis patients as compared to that of 24.73 ± 5.20 I the age-matched healthy controls.

In group 2, the DBH activity was also found to be decreased in the arsenicosis patints as compared to the control group. The DBH activity was also decreased in the serum of older arsenicosis patients as compared to that in the age-matched control group. The results focused on the issues that arsenicosis might effect the neuronal activities seriously, leading to the onset of various neurological disorders and the group 1 arsenicosis patients are prone to this arsenicosis disease.

Ascorbic acid is coenzyme for the activity of DBH and its contents were decreased in arsenicosis patients as compared to the healthy control groups. The DBH activities were also decreased, so these relationships were clear, but ascorbic acid is very unstable and is a strong reducing agent, so the specific role of ascorbic acid as coenzyme of DBH enzyme in arsenicosis is not clear.

The protein levels were decreased in all groups of the arsenicosis serum samples as compared to the age-matched healthy controls. The results are in agreements to many of our studies on protein patterns in arsenicosis serum and tissues of mammals. The details of our protein results on the arsenicosis samples will be published else where, but in a nut shell it can be said that in arsenic osis some serum proteins of smaller molecular weights of the ranges of 20–100 KDa are either reduced greatly or they are totally abolished missed in the serum or tissues, leading to the on set of many diseases, on the other hand, some lagre M.W. proteins are accumulated in the tissues or serum.

Zinc is an important micronutrient and also a cofactor of many enzymes, such as lactate dehydrogenase, arginase, etc. Zinc content was found to be decreased in patients as compared to the age-matched controls. In this type of condition usually dopamine levels are significantly higher in zinc deficient animals. This suggests the behavioral changes may be in part catecholamine related (25).

The glucose concentrations in the serum of arsenic patients were slightly increased as compared to age matched controls, but did not vary significantly which is in accordance with the study carried out by Rahman *et al*. that the arsenic exposure is a risk factor for diabetes mellitus (26). The another important parameter is vitamin A which is essential for growth and also for normal function of retina and development of epithelial surfaces. The vitamin A has many other vital functions of the body. Many transport proteins and retinol binding proteins are affected by arsenicosis.

The results of the present studies were concluded as follows: The DBH activity was found to be decreased in the serum of arsenicosis patients. The levels of ascorbic acid and copper (cofactors of DBH) and zinc were found to be decreased in the serum of these patients. The total protein level was decreased, but glucose level was increased in these serum samples. These data would be useful to the health planners, doctors and researchers on arsenicosis.

The situation of arsenicosis in Bangladesh is terrible. The Government has taken initiativeness for the urgent mitigation of arsenic poisoning. Now the people has become careful about drining water, specially that from the shallow-tube wells. But most of the affected people of arsenicosis are still beyond the medical treatment in Bangladesh (27).
